# Community-acquired Acinetobacter radioresistens bacteremia in an HIV-positive patient.

**DOI:** 10.3201/eid0706.010621

**Published:** 2001

**Authors:** P Visca, A Petrucca, P De Mori, A Festa, E Boumis, A Antinori, N Petrosillo

**Affiliations:** Dipartmento di Biologia, Università di Roma, Rome, Italy. visca@bio.uniroma3.it

## Abstract

We describe the first case of community-acquired bacteremia caused by Acinetobacter radioresistens; the patient was a 32-year-old HIV-positive neutropenic woman. Ambiguous Gram staining and poor biochemical reactivity of blood culture isolates misguided early diagnosis and therapy. Bacterial identification was based on 16S rDNA sequence analysis. A. radioresistens can be considered as a cause of opportunistic infection in immunodeficient patients.


### Community-Acquired *Acinetobacter radioresistens* Bacteremia in an HIV-Positive Patient

**Paolo Visca,*† Andrea Petrucca,‡ Patrizia De Mori,† Anna Festa,† Evangelo Boumis,† Andrea Antinori,† and Nicola Petrosillo†**
*Università di Roma Tre, Rome, Italy; †Istituto Nazionale di Malattie Infettive "Lazzaro Spallanzani," Rome, Italy; and ‡Istituto Superiore di Sanità, Rome, Italy

We describe the first case of community-acquired bacteremia caused by *Acinetobacter radioresistens*; the patient was a 32-year-old HIV-positive neutropenic woman. Ambiguous Gram staining and poor biochemical reactivity of blood culture isolates misguided early diagnosis and therapy. Bacterial identification was based on 16S rDNA sequence analysis. *A. radioresistens* can be considered as a cause of opportunistic infection in immunodeficient patients.

Members of the genus *Acinetobacter* are described as gram-negative, strictly aerobic diplococcoid rods that are oxidase negative and catalase positive (1). The genus includes at least 19 genomic species, defined on the basis of DNA relatedness criteria (2), which are ubiquitous in nature and have become increasingly responsible for a range of systemic infections in critically ill and immunocompromised patients (3). Genospecies 1 (*A. calcoaceticus*), 2 (*A. baumannii*), 3, and 13TU, classified as the *Acb complex*, are prevalent in nosocomial pneumonia and bacteremia but rarely colonize healthy persons (3,4). Genospecies 8/9 (*A. lwoffii*), 15BJ, and 12 (*A. radioresistens*) constitute part of the normal skin microflora but are seldom associated with human infections (5).

*Acinetobacter* spp. are responsible for 1%-2% of nosocomial bloodstream infections (4,6), in which *A. baumannii* represents the most commonly isolated species (3,7). Few *Acinetobacter* bacteremias are community acquired (8). The respiratory system and vascular devices are the main portals for entry of *Acinetobacter* into the bloodstream of critically ill persons (9). Secondary bloodstream invasion, resulting from dissemination of the bacterium from covert colonization sites, can also be considered when evidence of primary infection is missing (10). The outcome of *Acinetobacter* bacteremia is usually benign, with the prognosis depending on the severity of underlying disease(s) and the efficacy of antibiotic therapy (7-10).

In most clinical microbiology laboratories, identification of *Acinetobacter* cannot routinely be achieved at the genospecies level because commercial identification systems are substantially deficient and poorly discriminatory in distinguishing these organisms. This implies that local data on the prevalence of individual species in human infections should be interpreted cautiously unless supported by DNA-based taxonomy. Here we report a case of community-acquired *A. radioresistens* bacteremia in an HIV-positive patient, in which the causative agent was identified by means of 16S ribosomal DNA (rDNA) sequencing.

Address for correspondence: Paolo Visca, Dipartimento di Biologia, Università di Roma Tre, Viale G. Marconi 446, 00146 Rome, Italy; fax: +39-06-5517-6321; e-mail: visca@bio.uniroma3.it

### The Study

In January 2000, a 32-year-old HIV-positive woman was admitted to the National Institute for Infectious Diseases "L. Spallanzani," Rome, with a 10-day history of fever, productive cough, headache, rhinitis, and muscular pain. She tested HIV positive in 1993, citing heterosexual risk factors. In December 1999, she had HIV viremia of <80 copies/mL and a $CD4^+$ cell count of 309/mm$^3$. She had never taken antiretroviral therapy and had not been on antibiotic treatment in the previous 6 months.

The patient's recent history included chronic left suppurative otitis media with ear drainage and recurrent attacks of headache. One week before admission she had undergone computerized tomography scans of the brain, with contrast infusion; the scans were normal.

On admission the patient had fever (37.8°C), pallor, headache, left ear pain, and hearing loss. Lung examination revealed sparse crackles, but the chest radiograph was normal. Laboratory values were significant for leukopenia, with a leukocyte count of 2.5x10$^3$/mm$^3$ (normal range 4.3-10.8x10$^3$/mm$^3$), and neutropenia (1.0x10$^3$/mm$^3$; normal range 1.4-7.5x10$^3$/mm$^3$). C-reactive protein (CRP) was 2.1 mg/L (normal values <6 mg/L), erythrocyte sedimentation rate (ESR) 66 mm in the first hour (normal values <15 mm per hour), and platelet count 118x10$^3$/mm$^3$ (normal range 140-440/mm$^3$). The urine was normal, as were electrolytes, glucose, hemoglobin, and creatinine. X-ray examination of the sinuses revealed thickening of the right mucous membranes. Otoscopy revealed chronic left middle ear disease with acute inflammation. Two blood cultures, taken 3 and 6 hours after admission, were negative.

After 2 days in hospital, the patient returned home against the advice of the physicians. At home she was feverish and had continuous headache and reoccurence of left ear pain. One week later she was readmitted with fever (39.1°C), leukopenia (2.5x10$^3$/mm$^3$), neutropenia (0.7x10$^3$/mm$^3$), higher CRP levels (3.2 mg/L), and accelerated ESR (70 mm per hour). Physical examination showed no substantial changes compared with one week earlier. Because of the patient's symptoms and the results of otoscopy, a diagnosis of chronic middle ear disease was made, and she was treated empirically with intravenous ceftriaxone (2 g once a day) and gentamicin (80 mg three times a day).

Three days later, five of six blood cultures taken at 3-hour intervals during the first day of her second admission yielded visible bacterial growth. Gram staining of blood-free supernatants from all positive cultures was interpreted as showing a homogeneous smear of gram-positive diplococci and was used as the inoculum of Sceptor gram-positive Breakpoint/ID panels (Becton Dickinson, Franklin Lakes, NJ). Individual isolates from all five positive blood cultures showed an identical antibiotic-susceptibility pattern; they were resistant to penicillin, oxacillin, amoxicillin/clavulanate, clarithromycin, clindamycin, chloramphenicol, erythromycin, vancomycin, and teicoplanin, but sensitive to aminoglycosides, carbapenems, cephalosporins, ciprofloxacin, cotrimoxazole, and tetracycline. Bacterial identification-could not be achieved because of lack of biochemical reactivity of the strain. Previous antibiotic therapy was discontinued, and intravenous ciprofloxacin (400 mg twice a day) was begun for 2 weeks.

All blood culture isolates grew vigorously at 37°C on both chocolate agar and Columbia agar base supplemented with 5% (vol/vol) sheep blood, giving similarly smooth, opaque, nonhemolytic colonies. Tiny colonies appeared on eosin-methylene blue (EMB)-lactose agar after 36-48 hours' incubation. The presence of gram-positive coccobacillary forms, mostly organized in pairs, was confirmed for primary isolates. Growth was not detected on either mannitol salt agar or D-coccosel agar or, under anaerobic conditions, on Columbia agar base. Catalase and oxidase reactions were positive and negative, respectively. Infection by *Alloiococcus otitidis* was initially suspected, but specific biochemical tests and antibiotic susceptibility data argued against this hypothesis (data not shown).

Bacterial identification was achieved by means of 16S ribosomal DNA (rDNA) sequence analysis. Genomic DNA was extracted from each of the five isolates with a commercial kit (Quiagen genomic-tip, Qiagen Inc., Valencia, CA), and polymerase chain reaction (PCR) amplification was performed with universal primers annealing at the extreme 5' and 3' ends of the eubacterial 16S rDNA (encompassing nucleotides 9-27 and 1492-1512 relative to the *Escherichia coli* 16S rDNA sequence, International Union for Biochemistry [IUB] nomenclature) (2). The 16S rDNA amplicon was purified with the QUAquick PCR purification kit (Qiagen) and partially sequenced on one strand from the 5'-end using an ABI PRISM 377 (PE Applied Biosystems, Foster City, CA) automated sequencer and dye-labeled dideoxy chain-terminator chemistry (Dye Terminator Cycle sequencing Ready Reaction Kit, Applied Biosystems Inc.). Identical partial sequences were obtained for all the five amplicons analyzed, corresponding approximately to nucleotides 60-500 of the *E.coli* 16S rDNA gene sequence. Comparative BLAST software (version 2.0, National Center for Biotechnology Institute, http://www.ncbi.nlm.nih.gov/BLAST/) analysis with entries available at the EMBL, GenBank, and Ribosomal Data Project (http://www.cme.msu.edu/RDP/) databases retrieved an optimum alignment (99.4% identity) with the 16S rDNA of the *A. radioresistens* type strain M17694 (GenBank sequence accession number Z93445; ref. 2), and an excellent match with the published *A. radioresistens* 16S rDNA signature regions (Table). The sequence within the hypervariable helix 6 showed a G/A mismatch at position 75, compared with the published *A. radioresistens* sequence (2). However, the same single-base difference was found in the corresponding 16S rDNA signature of the partial sequence recently deposited under the accession number AJ247210, corresponding to *A. radioresistens* LMG 10614 (Harmsen D, Singer C, unpub. data).

Biochemical identification was repeated with the Sceptor gram-negative Breakpoint/ID and API 20NE panels. Both systems misidentified the organism as *A. lwoffi* (Sceptor and API codes were 0000000 and 0000032, respectively), although the combined results of both biochemical and assimilation tests were compatible with the identification as *A. radioresistens*.

Ten days after beginning the course of ciprofloxacin, the patient improved symptomatically, her temperature subsided, and serologic markers of inflammation declined (CRP and ESR values were 0.9 mg/L and 25 mm, respectively). She was discharged from hospital 4 days later, and she had no recrudescence of otitis or bacteremia in a 3-month follow-up period.

Possible sources of contamination were retrospectively investigated and ruled out. Infection control procedures in the unit were reviewed, and sterility control of 24 randomly sampled blood culture bottles from the same batch gave negative results. Moreover, no other strains similar to *A.radioresistens* were isolated in our institute from November 1999 to March 2000.

### Conclusions

To our knowledge, this is the first description of *A.radioresistens* causing community-acquired bacteremia. We speculate that systemic disease developed in our patient as a result of local infection; the combination of neutropenia and her impaired immunologic condition due to HIV infection made her susceptible to the infection.

Paranasal sinuses and the middle ear are potential reservoirs from which bacteria, including *Acinetobacter* spp., can enter the bloodstream; otitis media and sinusitis often precede bacteremia in predisposed patients (11 and references therein). Thus, we speculate that the left middle ear was the most likely portal for the entry of *A. radioresistens* into the bloodstream of the patient, although other sites cannot be ruled out. The history of recurrent episodes of ear drainage and the rapid remission of signs and symptoms following targeted antimicrobial therapy point to the middle ear infection as a plausible source for the systemic spread of *A. radioresistens*. Unfortunately, no clinical specimen was obtained for culture from the middle ear of the patient to confirm the diagnosis.

A Gram stain of bacteria from positive blood cultures is considered to be an important guide for the etiologic diagnosis and initial antibiotic choice. However, *Acinetobacter* spp. are known for being extremely resistant to decolorization (1), and diagnostic errors due to misinterpretation of well-prepared Gram stains have been reported (12). In our case, the gram-positive appearance of primary cultures of

Table. Sequence motifs of the variable regions for *Acinetobacter* 16S rRNAs, encompassing positions 70-101 (helix 6) and 453-477 (helix 18)[a]

| DNA group | Helix 6 variable region | Helix 18 variable region |
|---|---|---|
| 1 | GGAAGGUUGCUUCGGUAACUGACCUA | GCUCUCUUAGUUAAUACCUAAGAUG |
| 2 | GGGAAGGUAGCUUGCUACCGGACCUA | CCUACUUUAGUUAAUACCUAGAGAU |
| 3 | AGAGAGGUAGCUUGCUACUGAUCUUA | GCUACUUUAGUUAAUACCUAGAGAU |
| 4 | GGAAGGGUACCUUGCUACCUAACCUA | GCUACUCUAGUUAAUACCUAGAGAU |
| 5 | AGAUGAGGUGCUUGCACCUUAUCUUA | GCUACUGAGACUAAUACUCUUGGAU |
| 6 | GGUGAUGUAGCUUGCUACAUUACCUA | GCUACCUAGACUAAUACUCUAGGAU |
| 7 | GGAGAGGUAGCUUGCUACCUAACCUA | GCUACUUGGAUUAAUACUCUAGGAU |
| 8 | GGAGAGGUAGCUUGCUACAUAACCUA | GCUACCGAGAUUAAUACUCUUGGAU |
| 9 | GGAAGNGUAGCUUGCUACAUAACCUA | GCUACCGAGAUUAAUACUCUUGGAU |
| 10 | GGGAGAUUGCUUCGCUAAUUGACCUA | GCUCUUUUGGUUAAUACCCAAGAUG |
| 11 | GGGAGAUUGCUUCGGUAACUGACCUA | CCUCUCUUGGUUAAUACCCAAGAUG |
| 12 | AUGAA**G**GUAGCUUGCUACUGGAUUCA | GCUACCUAGAUUAAUACUUUAGGAU |
| AJ247210 | AUGAA**A**GUAGCUUGCUACUGGAUUCA | GCUACCUAGAUUAAUACUUUAGGAU |
| AR | AUGAA**A**GUAGCUUGCUACUGGAUUCA | GCUACCUAGAUUAAUACUUUAGGAU |
| TU13 | GGGAAGGUAGCUUGCUACUGGACCUA | GCUACUCUAGUUAAUACCUAGGGAU |
| TU14 | GGAAGGGUAGCUUGCUACCUAACCUA | CCUACCUAGAUUAAUACUCUAGGAU |
| TU15 | GGAUAGGUUGCUUGCACUUGAUGCUA | GCUUACCUGGUUAAUACCUGGGAUA |
| CTTU13 | GGAGAGGUAGCUUGCUACUGAUCUUA | GCUACUUUAGUUAAUACCUAGAGAU |
| 1-3 | GNUGAUGGUGCUUGCACUAUCACUUA | GCUACUUUAGUUAAUACCUAGAGAU |
| BJ14 | GGAAGGUUGCUUCGGUAUCUGACCUA | GCUCUCUUAGUUAAUACCUAAGAUG |
| BJ15 | AGUUAUGGUGCUUGCACUAUGACUUA | GCUCUCUUAGUUAAUACCUAAGAUG |
| BJ16 | AGUGAUGGUGCUUGCACUAUCACUUA | GCUACUAGUACUAAUACUACUGGAU |
| BJ17 | AGUGAUGGUGCUUGCACUAUCACUUA | GCUCUCCUAGUUAAUACCUAGGAUG |

[a]Representative strains for each DNA group (1 to 12, TU13 to TU15, CTTU13, 1-3, BJ14 to BJ17) are those listed in ref. 2. The designations AJ247210 and AR refer to *A. radioresistens* LMG 10614 (genospecies 12) and to our isolate, respectively. Nucleotides in bold highlight the differences between members of genospecies 12.

*A.radioresistens* delayed bacterial identification, and it was not until the organism was later observed growing on EMB agar that an incorrect diagnosis was suspected. Cases of *A. radioresistens* infection may be underestimated because this species escapes routine detection by most commercially available microbiologic tests (*A. radioresistens* is not included in the Sceptor version 3.10 database and in the API 20NE analytic catalog, 6th edition, 1998). Bacterial identification based on 16S rDNA sequence analysis can be performed directly on monomicrobic blood cultures and can be completed within 36 hours at relatively low cost. This case highlights the power of this technique for the rapid and correct identification of *A. radioresistens*.

### Acknowledgments

We thank K. Towner for critical reading of this manuscript.

This work was supported by grants from the Italian Ministry of Health, Progetti Finalizzati, and Ricerca Corrente IRCCS, to PV and NP.

Dr. Visca is associate professor of microbiology in the Department of Biology, University of Roma Tre, and head of the Molecular Microbiology Unit of the National Institute for Infectious Diseases "Lazzaro Spallanzani," Rome. His research interests include the molecular epidemiology and pathogenesis of infections caused by gram-negative bacteria.

### References

1. Schreckenberger PC, von Graevenitz A. *Acinetobacter*, *Achromobacter*, *Alcaligenes*, *Moraxella*, *Methylobacterium*, and other nonfermentative Gram-negative rods. In: Murray PR, Baron EJ, Pfaller MA, Tenover FC, Yolken RH, editors. Manual of clinical microbiology. Washington: American Society for Microbiology; 1999. p. 539-71.
2. Ibrahim A, Gerner-Smidt P, Liesack W. Phylogenetic relationship of the twenty-one DNA groups of the genus *Acinetobacter* as revealed by 16S ribosomal DNA sequence analysis. Int J Syst Bacteriol 1997;47:837-41.
3. Bergogne-Bérézin E, Towner KJ. *Acinetobacter* spp. as nosocomial pathogens: microbiological, clinical and epidemiological features. Clin Microbiol Rev 1996;9:148-65.
4. Forster DH, Daschner FD. *Acinetobacter* species as nosocomial pathogens. Eur J Clin Microbiol Infect Dis 1998;17:73-7.

5. Berlau J, Aucken H, Malnick H, Pitt TL. Distribution of *Acinetobacter* species on skin of healthy humans. Eur J Clin Microbiol Infect Dis 1999;18:179-83.
6. NNIS System. National nosocomial surveillance (NNIS) system report, data summary from October 1986-April 1998, issued June 1998. Available at http://www.cdc.gov/ncidod/hip/NNIS/sar98net.PDF
7. Seifert H, Strate A, Schulze A, Pulverer G. Bacteremia due to *Acinetobacter* species other than *Acinetobacter baumannii*. Infection 1994;22:379-85.
8. Tilley PAG, Roberts FJ. Bacteremia with *Acinetobacter* species: risk factors and prognosis in different clinical settings. Clin Infect Dis 1994;18:896-900.
9. Seifert H, Strate A, Pulverer G. Nosocomial bacteremia due to *Acinetobacter baumannii*: clinical features, epidemiology, and predictors of mortality. Medicine 1995;74:340-9.
10. Cisneros JM, Reyes MJ, Pachòn J, Becerril B, Caballero FJ, Garcìa-Garmendìa JL, et al. Bacteremia due to *Acinetobacter baumannii*: epidemiology, clinical findings, and prognostic features. Clin Infect Dis 1996;22:1026-32.
11. Bert F, Lambert-Zechovsky N. Sinusitis in mechanically ventilated patients and its role in the pathogenesis of nosocomial pneumonia. Eur J Clin Microbiol Infect Dis 1996;15:533-44.
12. Goodhart GL, Abrutyn E, Watson R, Root RK, Egert J. Community-acquired *Acinetobacter calcoaceticus* var. *anitratus* pneumonia. JAMA 1977;238:1516-18.
